# Effect of Dietary Copper on Intestinal Microbiota and Antimicrobial Resistance Profiles of *Escherichia coli* in Weaned Piglets

**DOI:** 10.3389/fmicb.2019.02808

**Published:** 2019-12-17

**Authors:** Yiming Zhang, Jian Zhou, Zhenglin Dong, Guanya Li, Jingjing Wang, Yikun Li, Dan Wan, Huansheng Yang, Yulong Yin

**Affiliations:** ^1^Hunan International Joint Laboratory of Animal Intestinal Ecology and Health, Laboratory of Animal Nutrition and Human Health, College of Life Sciences, Hunan Normal University, Changsha, China; ^2^Hunan Province Key Laboratory of Animal Nutritional Physiology and Metabolic Process, Key Laboratory of Agro-ecological Processes in Subtropical Region, National Engineering Laboratory for Pollution Control and Waste Utilization in Livestock and Poultry Production, Institute of Subtropical Agriculture, Chinese Academy of Sciences, Changsha, China

**Keywords:** copper, piglet, microbial community, *Escherichia coli*, antimicrobial resistance

## Abstract

Copper is an essential microelement for animals, and not only it has been used as a feed additive at pharmacological doses in swine production to improve growth performance, but it also has an effect on intestinal microbes by enhancing host bacterial resistance. However, there are few reports on the effects of pharmacological doses of copper on intestinal microorganisms and the antimicrobial resistance profiles of pathogenic bacteria, such as *Escherichia coli*, in pigs. Therefore, this study aimed to investigate the effects of pharmacological doses of copper on the microbial communities in the hindgut and the antimicrobial resistance profiles of *E. coli* in weaned piglets. Twenty-four healthy weaned piglets aged 21 ± 1 days and with an average weight of 7.27 ± 0.46 kg were randomly divided into four groups. The control group was fed a basal diet, while the treatment groups were fed a basal diet supplemented with 20, 100, or 200 mg copper/kg feed, in the form of CuSO_4_. Anal swabs were collected at 0, 21, and 42 days of the trial, and *E. coli* was isolated. Meanwhile, the contents of the ileum and cecum from the control and 200 mg copper/kg feed groups were collected at 21 and 42 days for microbial community analysis and *E. coli* isolation. All isolated *E. coli* strains were used for antimicrobial resistance profile analysis. A pharmacological dose of copper did not significantly change the diversity, but significantly affected the composition, of microbial communities in the ileum and cecum. Moreover, it affected the microbial metabolic functions of energy metabolism, protein metabolism, and amino acid biosynthesis. Specifically, copper treatment increased the richness of *E. coli* in the hindgut and the rates of *E. coli* resistance to chloramphenicol and ciprofloxacin. Moreover, the rate of *E. coli* resistance to multiple drugs increased in the ileum of pigs fed a pharmacological dose of copper. Thus, a pharmacological dose of copper affected the composition of the microbial community, increased the antimicrobial resistance rates of intestinal *E. coli*, and was most likely harmful to the health of piglets at the early stage after weaning.

## Introduction

Copper (Cu) is an essential trace element for animals, with many biological functions, including iron metabolism, immunity, protection from oxidative stress, and improvement in the activity of digestive enzymes (Gipp et al., 1967; Harris, 1991; Amachawadi et al., 2010; Braune et al., 2010; Huang et al., 2015). Feeding weaned piglets a high Cu diet, usually at pharmacological doses (150–250 mg Cu/kg feed) as CuSO_4_, Cu-AA, or Cu hydroxychloride, may improve their average daily weight gain and feed conversion ratio and reduce the frequency of diarrhea (Smith et al., 1997; Cromwell et al., 1998; Hill et al., 2000; Perez et al., 2011; Bikker et al., 2016; Espinosa et al., 2017).

However, as the Cu requirement of piglets is 4–6 mg Cu/kg feed (Okonkwo et al., 1979), the excess Cu results in an increase in the Cu content of pig slurry and, consequently, an accumulation of Cu in soils, which poses a high environmental risk (Jondreville et al., 2002). Meanwhile, excess amounts of Cu in the gut can affect the intestinal microbial community and result in reduced counts of streptococci, ureolytic bacteria, and total anaerobes in the feces (Fuller et al., 1960; Kellogg et al., 1966; Varel et al., 1987). Recently, it has been found that feeding piglets with 300 mg Cu/kg feed affected the abundance of *Clostridium genera* and decreased the relative abundance of butyrate-producing bacteria, such as *Acidaminococcus*, *Coprococcus*, and *Roseburia* in fecal microbiota (Zhang et al., 2019). It also altered the gut microbiota and decreased the counts of *Lactobacilli* and Enterobacteriaceae in the cecum and ileum of weaned piglets (Ole et al., 2005; Mei et al., 2010). However, the effects on gut microbiota composition and function when pharmacological amounts of Cu are added to feed have not been thoroughly investigated.

In pigs, enteric bacteria have been shown to develop Cu resistance by transferable tcrB in enterococci (Hasman and Aarestrup, 2005) or by the multicopper oxidase, PcoA, and the metal sponge, PcoE, in enterobacteria (Djoko et al., 2008; Zimmermann et al., 2012). Resistance to Cu in bacteria, particularly in enterococci, is often associated with resistance to antimicrobial drugs, such as macrolides and glycopeptides (Hasman et al., 2006; Yazdankhah et al., 2014), while Cu resistance in *Escherichia coli* is negatively associated with both tetA and bla (CMY-2) (Agga et al., 2014). Nevertheless, the potential connection between Cu resistance and antimicrobial drug resistance, particularly in the enterobacterium, *E. coli*, has not been fully elucidated. Therefore, the objective of the present study was to investigate the effects of different doses of Cu, including a pharmacological dose, on hindgut microbiota diversity, composition, and metabolic function in weaned piglets after 3 and 6 weeks of supplementation. Based on the results of microbiota analysis, *E. coli* was isolated from feces at 0, 21, and 42 days of the feeding period, and the drug resistance profile of the isolates was assessed for the first time.

## Materials and Methods

### Animals, Feeding, and Sampling

Animal experiments were approved by the Animal Protocol Review Committee of the Institute of Subtropical Agriculture, Chinese Academy of Sciences (Changsha, China), and all experiments were performed according to the institutional animal welfare requirements. Forty-eight healthy weaned piglets, aged 21 ± 1 days (8.26 ± 0.17 kg), were randomly assigned to four groups, with six barrows and six females in each group. The piglets were housed individually and given free access to drinking water and food. The control group was fed a basal diet (Table 1), and the treatment groups were fed a basal diet supplemented with 20, 100, or 200 mg Cu/kg feed, as CuSO_4_⋅5H_2_O, for 6 weeks. Anal swabs were collected from piglets at days 0, 21, and 42 of the experimental period, for *E. coli* isolation and antimicrobial drug resistance detection. At days 21 and 42, piglets (*n* = 6) in the control and 200 mg Cu group were anesthetized with Zoletil (15 mg/kg BW, i.m.) and euthanized. The ileal and cecal contents were collected under sterile conditions, using a 15-ml sterile conical centrifuge tube and submitted for 16S rRNA sequencing, *E. coli* isolation, and antimicrobial drug resistance evaluation.

**TABLE 1:** Formulation of basal diet (air-dry basis, %).

**Ingredient**	**Content (%)**	**Analyzed nutrient levels^a^**	**Content**
Corn	25	Dry matter (%)	87
Extruded corn	34	Digestible energy (kcal/kg)	3.40
Soymeal	2.5	CP (%)	20.00
Fermented soymeal	8	Ash (%)	7.84
Extruded soybean	5	Ether extract (%)	3.55
Fish meal	10	NDF (%)	22.16
Whey powder	4	ADF (%)	11.75
Glucose	5	Ca (%)	0.80
L-Thr (≥97.5%)	0.45	Total P (%)	0.45
L-Trp (≥98.0%)	0.05	Cu (control)	5.09 mg/kg
DL-Met (≥98.5%)	0.2	Cu (20 mg/kg)	25.68 mg/kg
L-Lys⋅HCl (≥98.5%)	0.8	Cu (100 mg/kg)	81.80 mg/kg
Soybean oil	1.1	Cu (200 mg/kg)	176.58 mg/kg
Sodium chloride (≥91.0%)	0.74		
Calcium carbonate	0.4		
Calcium hydrophosphate	0.28		
Antioxidant	0.08		
Citric acid	1.3		
Mildew preventive	0.1		
Premix^b^	1		

*^*a*^Feed analyses were performed according to the AOAC guidelines (2004). ^*b*^The vitamin–mineral premix provided the following per kilogram of feed: 100 mg of antioxidant, 100 mg of preservatives, 100 mg of choline chloride, 6,000 IU of vitamin A, 3,000 IU of vitamin D_3_, 20 IU of vitamin E, 1.8 mg of vitamin K_3_, 2.0 mg of thiamine, 6.0 mg of riboflavin, 4.0 mg of pyridoxine, 0.02 mg of vitamin B_12_, 26.0 mg of niacin, 18.0 mg of pantothenic acid, 3.2 mg of folic acid, 0.4 mg of biotin, 100 mg of Fe as FeSO_4_⋅ 7H_2_O, 100 mg of Zn as ZnSO_4_⋅H_2_O, 50 mg of Mn as MnSO_4_⋅H_2_O, 1.2 mg of I as KI, and 0.30 mg of Se as Na_2_SeO_3_.*

### 16S rDNA Sequencing

The ileal and cecal contents from piglets in the control (without Cu supplementation) group and the group receiving a pharmacological dose of Cu (200 mg Cu/kg feed, *n* = 6 per treatment) were collected for microbiota analysis, as described previously (Zhou et al., 2018; Xiong et al., 2019). Briefly, total bacterial DNA was extracted using a QIAamp DNA Stool Mini Kit (Qiagen, Hilden, Germany), and the V3–V4 hypervariable region of the microbial 16S rRNA genes were sequenced using the primers, 341F: 5′-CCTAYGGGRBGCASCAG-3′ and 806R: 5′-GGACTACNNGGGTATCTAAT-3′; Illumina adaptors; and molecular barcodes. Libraries were prepared using a TruSeq DNA PCR-Free Sample Preparation Kit (Illumina Inc., San Diego, CA, United States) and were assessed using a Qubit 2.0 Fluorometer (Thermo Scientific, Madison, WI, United States) and a Bioanalyzer 2100 system (Agilent Technologies, Santa Clara, CA, United States). Libraries were sequenced on a HiSeq 2500 platform (Illumina Inc., San Diego, CA, United States). Raw 16S rDNA sequences were assembled using the QIIME (v1.9.0) and FLASH software packages. Operational taxonomic units (OTUs) were analyzed using UPARSE (v7.0.1001), and high-quality sequences were aligned against the SILVA reference database 1 and clustered into OTUs at a 97% similarity level using the UCLUST algorithm 2. Each OTU was assigned to a taxonomic level using the Ribosomal Database Project Classifier program v2.203. The assembled sequences were then submitted to the NCBI Sequence Read Archive (No. PRJNA551517) for open access.

### *E. coli* Isolation and Identification

Samples were collected with sterile swabs, and the swab sample was immediately inserted into a 15-ml sterile centrifuge tube containing 5 ml of phosphate-buffered saline. Then, 10 μl of each sample was swabbed onto MacConkey agar and incubated at 37°C for 18–24 h. Three red- or pink-colored colonies were picked per sample and inoculated on eosin methylene blue agar plates at 37°C for 18–24 h to obtain pure cultures. A single colony with a black metallic luster was picked and cultured in Luria–Bertani (LB) liquid medium at 37°C for 8–12 h. Bacterial smears were from the LB liquid culture, and Gram staining and microscopic examination were performed as described (Feyzioglu et al., 2014; Usaini et al., 2015). LB liquid cultures containing Gram-negative bacilli were swabbed onto eosin methylene blue agar plates and incubated at 37°C for 18–24 h. A single colony with a black metallic luster was picked, inoculated on a nutrient agar plate, and cultured at 37°C for 12–18 h. Single colonies from the nutrient agar plate were inoculated in biochemical identification tubes (Hangzhou Microbial Reagent Co., Ltd., Hangzhou, China) for lactose fermentation, and indole and citrate utilization tests, according to the manufacturer’s instructions. Isolates with a Lac+/Ind+/Cit− phenotype were identified as *E. coli*. Finally, the 16S rRNA gene of isolated *E. coli* was amplified by PCR. DNA extraction and PCR amplification were performed as previously described (Blyton et al., 2013). Three oligonucleotide primers, 16E1, 16E2, and 16E3, were designed and used in the PCR assays for the identification of *E. coli* isolates, as previously described (Tsen et al., 1998). The sequences and locations of these primers are shown in Supplementary Table S1. PCR products were analyzed by gel electrophoresis through a 2% agarose gel, as previously described (Tsen et al., 1998) and sequenced by Sangon Biotech Co., Ltd. (Shanghai, China). Homology analysis between the amplified sequences and the *E. coli* 16S rRNA gene sequences published in GenBank was performed to confirming that the isolates were *E. coli*. Repetitive element PCR with both ERIC and (CGG)4 primers was also used to discriminate clonal isolates from the same samples (Adamus-Bialek et al., 2009). Isolates producing the same banding pattern were considered to be the same strain.

### Drug-Resistant Phenotype Identification

After PCR validation, the *E. coli* suspensions were subjected to antimicrobial susceptibility testing on Müller–Hinton agar using the Kirby–Bauer disk diffusion method, in accordance with the Clinical and Laboratory Standards Institute guidelines, using *E. coli* ATCC 25922 as an internal quality control. The antimicrobial disks used for the test included ampicillin (10 μg/disk), amoxicillin/clavulanic acid (20/10 μg/disk), ceftriaxone (30 μg/disk), meropenem (10 μg/disk), aztreonam (30 μg/disk), gentamicin (10 μg/disk), trimethoprim/sulfamethoxazole (1.25/23.75 μg/disk), amikacin (30 μg/disk), and ciprofloxacin (CIP) (5 μg/disk) (Oxoid Ltd., Hampshire, United Kingdom).

### Statistical Analysis

All statistical analyses were performed using SPSS 20.0 software (SPSS Inc., Chicago, IL, United States). α and β diversity were analyzed using QIIME (v1.7.0). Data for microbial metabolic functions were analyzed by Student’s *t*-test only, due to minor changes in the microbiome data. The differences in isolation rates and antimicrobial resistance of *E. coli* among the groups were analyzed by Chi-square test. A *P* < 0.05 was considered statistically significant.

## Results

### Bodyweight and Growth Performance

The average bodyweights of the weaned piglets at 21 ± 1 days in the control and Cu-supplemented groups were 8.13 ± 0.22 and 8.40 ± 0.26 kg, respectively. There were no significant differences in average daily gain, average daily feed intake, or feed conversion ratio between the control and Cu-supplemented groups in early (1–3 weeks) and late postweaning stages (4–6 weeks, Table 2).

**TABLE 2:** The effect of Cu levels on the growth performance of piglets during the first 3 weeks and the second 3 weeks of feeding.

**Time**	**Growth performance^a^**	**Control**	**High Cu**	***P*-value**
	ADG (kg)	0.22 ± 0.01	0.25 ± 0.02	0.154
1–3 weeks	ADFI (g)	405.58 ± 14.62	444.57 ± 21.58	0.157
	FCR	1.86 ± 0.04	1.80 ± 0.09	0.559
	ADG (kg)	0.37 ± 0.02	0.42 ± 0.03	0.201
4–6 weeks	ADFI (g)	787.41 ± 34.66	805.18 ± 40.89	0.743
	FCR	2.12 ± 0.08	1.95 ± 0.09	0.177

*^*a*^ADG, average daily gain; ADFI, average daily feed intake; FCR, feed conversion ratio.*

### Microbiota Diversity in Intestinal Contents

**FIGURE 1:**
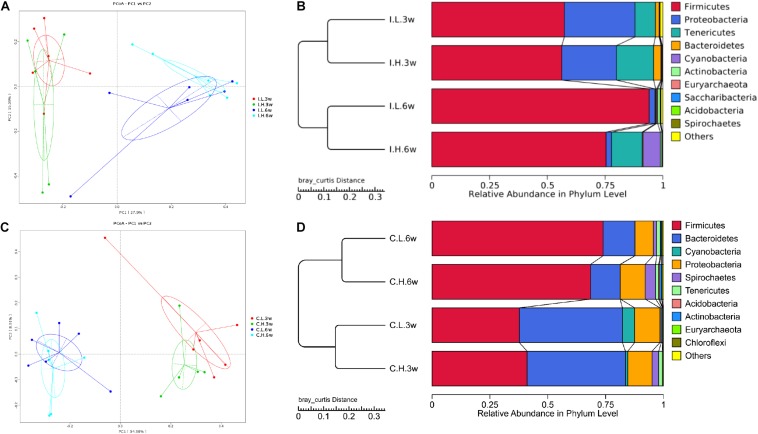
Principal coordinate analysis and unweighted pair-group method with arithmetic mean (UPMGA) cluster analysis of microbial communities in the ileum and cecum of weaned piglets after 3 and 6 weeks of feeding. **(A)** Principal coordinate analysis (PCoA) of microbial communities in the ileum, **(B)** UPMGA cluster analysis of microbial communities in the ileum, **(C)** PCoA of microbial communities in the cecum, and **(D)** UPMGA cluster analysis of microbial communities in the cecum. I, ileal microbiota; C, cecal microbiota; L, control group; H, Cu-supplemented group; 3w, 3 weeks of feeding after weaning; 6w, 6 weeks of feeding after weaning.

There were no significant differences between treatments for the indices of α diversity, including observed_species, Shannon, Simpson, Chao1, ACE, and PD_whole_tree indices, of ileal (Supplementary Table S2) and cecal microbiota (Supplementary Table S3). In addition, the weighted principal coordinate analysis plots of ileal (Figure 1A) and cecal microbiota (Figure 1C) verified that there were few differences in microbial communities between Cu treatments.

### Microbiota Composition of Intestinal Contents

**FIGURE 2:**
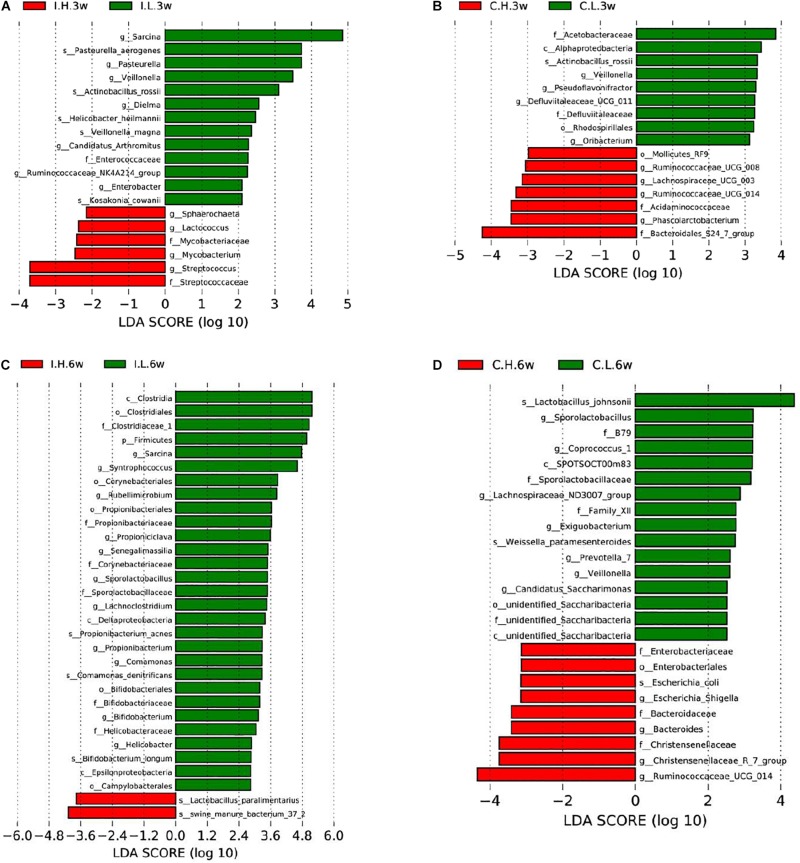
Significantly different compositions of the ileal and cecal microbiota between control and Cu-supplemented groups determined by linear discriminant analysis (LDA) effect size analysis after 3 and 6 weeks of feeding in weaned piglets. Significantly different compositions of the ileal **(A)** and cecal **(B)** microbiota after 3 weeks of feeding and significantly different compositions of the ileal **(C)** and cecal **(D)** microbiota after 6 weeks of feeding. I, ileal microbiota; C, cecal microbiota; L, control group; H, Cu-supplemented group; 3w, 3 weeks of feeding after weaning; 6w, 6 weeks of feeding after weaning.

Based on an unweighted pair-group method with arithmetic mean analysis, Firmicutes, Proteobacteria, Tenericutes, and Bacteroidetes were found to be the dominant bacteria in the ileal microbiome (Figure 1B), while Firmicutes, Bacteroidetes, Cyanobacteria, Proteobacteria, and Spirochaetes were the dominant bacteria in the cecal microbiome (Figure 1D). Furthermore, the linear discriminant analysis effect size method was used to determine the variation in microbial composition between groups. Minimal changes were observed after short-term feeding (3 weeks), with differences in abundance of the genera *Sarcina*, *Pasteurella*, *Veillonella*, *Streptococcus*, *Mycobacterium*, *Lactococcus*, and *Sphaerochaeta* in the ileal microbiome (Figure 2A) and in the family, Acetobacteraceae, and the genera, *Veillonella*, *Pseudoflavonifractor*, Defluviitaleaceae, *Oribacterium*, Ruminococcaceae (UCG008, 014), Lachnospiraceae (UCG003), and *Phascolarctobacterium* in the cecal microbiome (Figure 2B). Meanwhile, after a longer time of feeding (6 weeks) with Cu supplementation at a pharmacological dose, marked differences were found in the abundance of the family, Clostridiaceae, the phylum Firmicutes, the genus, *Sarcina*, *Lactobacillus paralimentarius*, and swine manure bacterium in the ileal microbiome (Figure 2C), and the genera, *Lactobacillus* and *Sporolactobacillus* and *E. coli* in the cecal microbiome (Figure 2D). A heat map of the 40 most different genera showed the taxonomic distributions among groups after 3 and 6 weeks of treatment (Supplementary Figure S1). Next, the impact of Cu treatment on aerobic, anaerobic, mobile element-containing, facultatively anaerobic, biofilm-forming, Gram-negative, Gram-positive, potentially pathogenic, and stress-tolerant bacteria were predicted via BugBase (Zhou et al., 2019). No significant differences were found to occur as a result of Cu treatment (Supplementary Figure S3).

### Predicted Metabolic Functions in the Gut Microbiota

**FIGURE 3:**
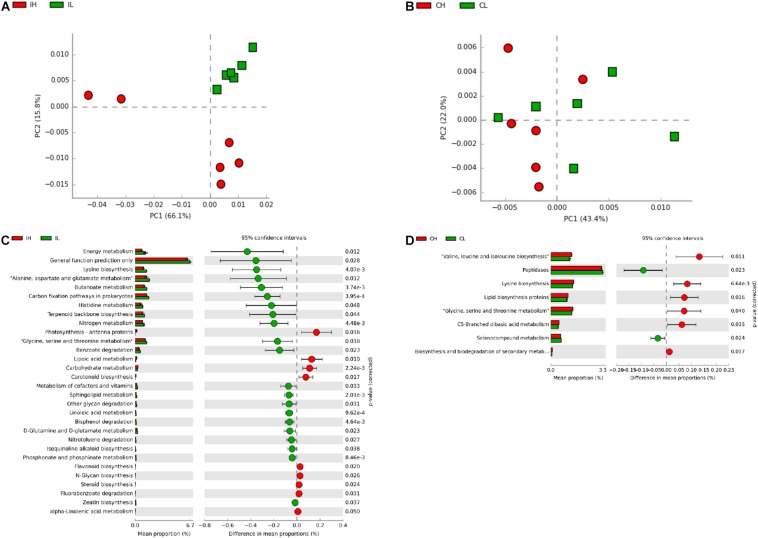
Analysis of functional differences in the ileal and cecal microbiota of piglets between control and Cu-supplemented groups after 6 weeks of feeding. Principal component analysis (PCA) of the functions of the ileal **(A)** and cecal **(B)** microbiota, significantly different functions of the ileal **(C)** and cecal **(D)** microbiota. IL, ileal microbiota in control group; IH, ileal microbiota in Cu-supplemented group; CL, cecal microbiota in control group; CH, cecal microbiota in Cu-supplemented group. Data were analyzed by Student’s *t*-test only, due to the minor changes in these microbiome data.

Subsequently, to further investigate the changes in the function of the gut microbiota, the metagenome was generated, based on 16S rDNA sequencing results, using Picrust. The Kyoto Encyclopedia of Genes and Genomes annotation results were then submitted for principal component analysis. Microbial metabolism-related pathways at Kyoto Encyclopedia of Genes and Genomes level 3 were specifically filtered. No obvious segregation of ileal and cecal microbiomes was found in the third week (Supplementary Figures S2A,B). Furthermore, a *t*-test showed that Cu supplementation (200 mg Cu/kg feed) increased the abundance of microbiota related to the transcriptional machinery and tuberculosis in the ileal microbiome, while an increase in the abundance of microbiota related to methane metabolism was observed in the cecal microbiome at the third week (Supplementary Figures S2C,D). An obvious segregation was found in the ileal microbiome in the sixth week (Figure 3A), but there was still no obvious segregation in the cecal microbiome at this time point (Figure 3B). Cu supplementation decreased the abundance of microbiota related to energy metabolism, lysine biosynthesis, amino acid metabolism (alanine, aspartate, glutamate, histidine, glycine, serine, and threonine), butanoate metabolism, carbon-fixation pathways in prokaryotes, terpenoid backbone biosynthesis, nitrogen metabolism, benzoate degradation, and the metabolism of cofactors and vitamins in the ileal microbiome (Figure 3C). Meanwhile, Cu supplementation increased the abundance of microbiota related to amino acid biosynthesis (valine, leucine, isoleucine, and lysine), lipid biosynthesis protein, amino acid metabolism (glycine, serine, and threonine), and C5-branched dibasic acid metabolism and decreased the abundance of microbiota related to peptidases in the cecal microbiome (Figure 3D).

### Antimicrobial Resistance Profiles of *E. coli* Isolates From Intestinal Contents

Based on microbial composition analysis, we further determined the antimicrobial resistance profiles of *E. coil* isolated from the ileal and cecal contents. Isolates that were resistant to three or more classes of antimicrobials were defined as multidrug-resistant strains. A total of 119 *E. coli* isolates from ileal (*n* = 50) and cecal contents (*n* = 69) were included in disk diffusion testing, with 20 ileal isolates and 34 cecal isolates from control pigs and 30 ileal isolates and 35 cecal isolates from pigs fed a pharmacological dose of Cu (Supplementary Table S4).

Of the 119 isolates, 10 (8.40%) were not resistant to any of the antimicrobial agents tested, 9 (7.56%) were resistant to one antimicrobial agent, and 21 (17.65%) were resistant to two antimicrobial agents. Furthermore, 79 (66.39%) were resistant to at least three antimicrobial agents, and 47 (39.50%) of these were isolated from pigs fed a pharmacological dose of Cu. In particular, seven isolates (5.88%) were resistant to more than six classes of antimicrobials, and six (5.04%) of these were isolated from pigs fed a pharmacological dose of Cu (Table 3). The antimicrobial resistance rates of *E. coli* in the ileum and cecum are summarized in Table 4. Resistance to CIP in ileal isolates and resistance to chloramphenicol (C) in cecal isolates were found to significantly respond to Cu dose.

**TABLE 3:** Number of antimicrobial multiresistant *E. coli* isolates in ileum and cecum.

	**Ileum**		**Cecum**		**Overall (*n* = 119)**
	**Control (*n* = 20)**	**High Cu (*n* = 30)**	**Control (*n* = 34)**	**High Cu (*n* = 35)**	
0	3	4	2	1	10
1	0	1	3	5	9
2	10	1	4	6	21
3	3	6	11	8	28
≥4 and ≤6	4	15	13	12	44
>6	0	3	1	3	7

**TABLE 4:** Antimicrobial resistance rates (95% CI) of *Escherichia coli* in ileum and cecum^1^.

**Antimicrobial agent^a^**	**Groups**	**Ileum *E. coli***	**Cecum *E. coli***
AMC	Control	0.00 (0.00, 16.84)	0.00 (0.00, 10.28)
	High Cu	6.67 (0.82, 22.07)	5.71 (0.70, 19.16)
	*P*-value	0.510	0.493
CRO	Control	10.00 (1.24, 31.70)	11.76 (3.30, 27.45)
	High Cu	20.00 (7.71, 38.57)	14.29 (4.81, 30.26)
	*P*-value	0.450	1.000
C	Control	75.00 (50.90, 91.34)	47.06 (29.78, 64.87)
	High Cu	80.00 (61.43, 92.29)	74.29 (56.74, 87.51)
	*P*-value	0.736	0.027
CN	Control	20.00 (5.73, 43.66)	41.18 (24.65, 59.30)
	High Cu	20.00 (7.71, 38.57)	28.57 (14.64, 46.30)
	*P*-value	1.000	0.318
CIP	Control	15.00 (3.21, 37.89)	35.29 (19.75, 53.51)
	High Cu	46.67 (28.34, 65.67)	25.71 (12.49, 43.26)
	*P*-value	0.032	0.440
AMP	Control	50.00 (27.20, 72.80)	73.53 (55.64, 87.12)
	High Cu	73.33 (54.11, 87.72)	71.43 (53.70, 85.36)
	*P*-value	0.134	1.000
ATM	Control	0.00(0.00, 16.84)	2.94 (0.07, 15.33)
	High Cu	13.33(3.76, 30.72)	8.5 7(1.80, 23.06)
	*P*-value	0.140	0.614
MEM	Control	0.00 (0.00, 16.84)	2.94 (0.07, 15.33)
	High Cu	0.00 (0.00, 11.57)	5.71 (0.70, 19.16)
	*P*-value		1.000
AK	Control	0.00 (0.00, 16.84)	5.88 (0.72, 19.68)
	High Cu	13.33 (3.76, 30.72)	14.29 (4.81, 30.26)
	*P*-value	0.140	0.428
SXT	Control	80.00 (56.34, 94.27)	94.12 (80.32, 99.28)
	High Cu	80.00 (61.43, 92.29)	91.43 (76.94, 98.20)
	*P*-value	1.000	1.000

*^1^The differences in antibiotic resistance of *E. coli* among groups were analyzed by Chi-square test. A *P* < 0.05 was considered statistically significant. ^*a*^AMP, ampicillin; AMC, amoxicillin/clavulanic acid; CRO, ceftriaxone; MEM, meropenem; ATM, aztreonam; CN, gentamicin; AK, amikacin; CIP, ciprofloxacin; C, chloramphenicol; SXT, trimethoprim/sulfamethoxazole.*

On the basis of these results, anal swabs were collected from four groups of pigs and used for *E. coli* isolation to investigate the development of resistance in response to Cu dose and feeding period. No significant differences were found in the rate of *E. coli* response to Cu dosage or feeding period (Supplementary Table S5). However, a dose-dependent effect on resistance to C and CIP was found at day 42 (*P* < 0.05, Table 5).

**TABLE 5:** Antimicrobial resistance rates (95% CI) of *Escherichia coli* from anal swabs^1^.

**Antibiotic^s^**	**Time (weeks)**	**Copper treatments (mg Cu/kg feed)**				***P*-value**
		**0**	**20**	**100**	**200**	
	0	53.33(26.59,78.73)	46.67(21.27,73.41)	53.33(26.59,78.73)	38.89(17.30,64.25)	0.812
C	3	53.33(26.59,78.73)	29.41(10.31,55.96)	60.00(32.29,83.66)	66.67(38.38,88.18)	0.153
	6	19.05(5.45,41.91)^a^	23.81(8.22,47.17)^a^	46.67(21.27,73.41)^a^	78.26(56.30,92.54)^b^	0.000
	0	29.41(10.31,55.96)	11.11(1.38,34.71)	40.00(16.34,67.71)	50.00(26.02,73.98)	0.063
CIP	3	20.00(4.33,48.09)	11.76(1.46,36.44)	26.67(7.79,55.10)	13.33(1.66,40.46)	0.692
	6	14.29(3.05,36.34)^a^	4.76(0.12,23.82)^a^	26.67(7.79,55.10)^ab^	30.43(13.21,52.92)^b^	0.046

*^1^The differences in antibiotic resistance of *E. coli* among groups were analyzed by Chi-square test. ^*a,b*^Means in a row without a common superscript letter differ significantly for the same period. ^*s*^CIP, ciprofloxacin; C, chloramphenicol.*

## Discussion

For many years, a pharmacological dose of Cu (150–250 mg/kg feed) has been added to animal diets to improve growth performance. However, as the Cu requirement of pigs is 4–6 mg/kg feed, the growth-promoting effect of dietary Cu is mostly attributed to the regulation of the composition and metabolism of the intestinal microbiota (Varel et al., 1987). Meanwhile, there are increasing concerns regarding the potential adverse effects of Cu accumulation on the intestinal microbiota, i.e., microbial drug resistance (Hasman and Aarestrup, 2005; Hasman et al., 2006; Djoko et al., 2008; Amachawadi et al., 2011; Zimmermann et al., 2012; Agga et al., 2014; Yazdankhah et al., 2014). The effects of Cu on the counts of coliforms and microbial drug resistance have previously been reported (Djoko et al., 2008; Zimmermann et al., 2012; Zhang et al., 2019). Thus, the present research focused on microbial diversity, composition, and function, as well as the potential risks of *E. coli* resistance in the intestine of piglets after pharmacological doses of Cu.

α and β diversity are important features of microbial communities. In our study, α diversity indices, including observed_species, Shannon, Simpson, Chao1, ACE, and PD_whole_tree indices were not affected by dietary Cu. Our results were consistent with those of a previous study of high dietary Cu levels (300 mg/kg) in suckling piglets (Zhang et al., 2019) but in contrast to previous studies reporting that high dietary Cu levels (100–200 mg/kg, as CuSO_4_) significantly affect microbial species in the ileac, cecal, and colonic chyme of piglets (Varel et al., 1987; Hojberg et al., 2005; Namkung et al., 2006; Mei et al., 2010). A possible reason for these differences is that the method used to investigate the microbial flora is inconsistent among the different studies. As most bacterial species in the gut are yet to be cultured, 16S rRNA gene sequencing is recommended for the analysis of gut microbial communities. In the present study, unweighted pair-group method with arithmetic mean analysis showed that Firmicutes, Bacteroidetes, and Proteobacteria were the most abundant phyla in the ileum and cecum, which is similar to previous findings in piglets (Xiong et al., 2019; Zhang et al., 2019). Linear discriminant analysis effect size analysis also showed that the differences included, but were not limited to, the phylum Firmicutes, the family, Clostridiaceae, and the genera, *Streptococcus*, *Lactococcus*, and Ruminococcaceae, which were found to be affected in previous studies in which Cu was supplemented at high levels (200–300 mg/kg) as CuSO_4_ (Fuller et al., 1960; Kellogg et al., 1966; Varel et al., 1987; Hojberg et al., 2005; Xia et al., 2005; Namkung et al., 2006; Mei et al., 2010; Wang et al., 2012; Song et al., 2013; Zhang et al., 2019). In addition, consistent with previous studies, the addition of high concentrations of Cu to the diet had no significant effect on the populations of aerobic, anaerobic, mobile element-containing, facultatively anaerobic, biofilm-forming, Gram-negative, Gram-positive, potentially pathogenic, or stress-tolerant bacteria (Varel et al., 1987; Hojberg et al., 2005; Mei et al., 2010; Zhang et al., 2019). Moreover, in accordance with the results of Zhang et al. (2019), carbohydrate metabolism by ileal microbes was significantly enhanced by high levels of dietary Cu.

Based on Picrust predictions, our study suggested that Cu supplementation affected the abundance of microbiota related to protein metabolism, amino acid synthesis and metabolism, and carbohydrate metabolism in the intestine. In accordance with this result, multiomics analysis showed that dietary Cu levels affected protein and carbohydrate metabolites and amino acid metabolism. The abundance of the genera, Lachnospiraceae and Ruminococcaceae, was positively correlated with energy metabolism pathways, and the abundance of the genus, *Streptococcus*, was negatively correlated with amino acid metabolism pathways (Zhang et al., 2019). Thus, the abundance of these species was also altered by Cu supplementation, resulting in effects on energy metabolism and amino acid biosynthesis and metabolism in piglets. In addition, we found that Cu supplementation affected the abundance of cecal microbiota related to lipid metabolism, suggesting that Cu may affect lipid metabolism in the cecum by regulating the abundance of the cecal microbiota.

Specially, our results showed that the abundance of *E. coli* in ileal and cecal contents tended to increase with high levels of dietary Cu at 3 weeks, and its abundance significantly increased in the cecum after 6 weeks of a pharmacological dose of Cu. However, there was no significant difference in the rate of isolation of *E. coli* from anal swabs. This discrepancy may be due to the high abundance of *E. coli* in rectum. These results were of great interest, since *E. coli* is the main diarrhea-causing opportunistic pathogen in piglets and also has high a rate of drug resistance. Therefore, *E. coli* was isolated at the beginning, middle, and end of the trial. As *E. coli* is present at a high abundance in the intestine and feces, three clones were isolated from all samples for drug resistance analysis.

The Cu resistance system in *E. coli* mainly consists of the *cut* and *pco* systems (Brown et al., 1992, 1995). Other Cu resistance systems, such as the CusCFBA efflux system (Franke et al., 2003), have also been reported. The absorption, efflux, and distribution of Cu are controlled by these systems to balance the content of Cu inside and outside the cell and avoid excessive Cu in the cell (Harris, 1991; Bull and Cox, 1994). Under conditions of high Cu concentration, intestinal Cu-resistant *E. coli* were selected, and thus, the resistance of *E. coli* to Cu increased, resulting in an improvement in the adaptability to high levels of Cu and an increase in the abundance of *E. coli*. However, some previous studies have indicated that Cu inhibits the growth of *E. coli* in *in vitro* culture experiments (Ding et al., 2010; Reyes-Jara et al., 2016). This difference may be explained by the fact that the *E. coli* strain used for *in vitro* culturing did not possess a Cu resistance system, while the strains of *E. coli* in the intestinal tract varied, with some being resistant to Cu.

Copper promotes the selection of antibiotic resistance in *E. coli* (Page, 2003). Hölzel et al. (2012) found that high levels of Cu in liquid pig manure promoted the resistance of *E. coli* to β-lactam antibiotics. Our study showed that the addition of high levels of Cu in the diet also contributed to the resistance of *E. coli* to CIP and C in both the intestine and feces. The mechanism responsible for this increased antibiotic resistance of *E. coli* after exposure to high levels of Cu was not clear. Previous studies have shown that the Cu-resistance gene (*tcrB*), the macrolides-resistance gene (*ERM*), and the glycopeptide-resistance gene (*vanA*) of *Enterococcus faecium* are located on the same plasmid. *E. faecium* isolates with tcrB have been shown to be resistant to macrolides (erythromycin) and glycopeptides (vancomycin) (Hasman and Aarestrup, 2002; Amachawadi et al., 2010, 2011, 2013). Therefore, Cu resistance may play a synergistic role in the development of antibiotic resistance, through coresistance, cross-resistance, or coregulatory resistance.

In addition, *E. coli* isolates in the present study were found to be highly multidrug resistant. A previous study has shown that >88% of *E. coli* isolates from pig feces are multidrug resistant, which is in agreement with the results of our study (Agga et al., 2014). In our study, of the 79 isolates (66.39%) that were resistant to at least three antimicrobial agents, 47 (39.50%) were from piglets treated with a pharmacological dose of Cu, and only 32 isolates (26.89%) were from piglets in the low-Cu group. In particular, six isolates from the group treated with a pharmacological dose of Cu were found to be resistant to six or more classes of antimicrobials, whereas only one isolate resistant to six or more classes of antimicrobials was found in the control group. Our results indicated that a high level Cu supplementation in the diet contributed to an increase in the rate of multidrug resistance of *E. coli*. The mechanism by which Cu promotes multiple drug resistance in *E. coli* remains unclear and should be the focus of future studies.

## Data Availability Statement

The datasets generated for this study can be found in the https://www.ncbi.nlm.nih.gov/sra/PRJNA551517.

## Ethics Statement

The animal study was reviewed and approved by the Animal Protocol Review Committee of the Institute of Subtropical Agriculture, Chinese Academy of Sciences.

## Author Contributions

YZ, DW, HY, and YY designed the study. YZ, JZ, ZD, GL, JW, and YL performed the animal feeding trial and sample analysis. YZ and DW wrote and revised the article.

## Conflict of Interest

The authors declare that the research was conducted in the absence of any commercial or financial relationships that could be construed as a potential conflict of interest.
