# Supplementary material for: Effect of Dietary Copper on Intestinal Microbiota and Antimicrobial Resistance Profiles of Escherichia coli in Weaned Piglets
Source: Front Microbiol. 2019 Dec 17;10:2808. doi: 10.3389/fmicb.2019.02808 (PMC6927916; doi:10.3389/fmicb.2019.02808)
Supplement: Supplementary file 1 [file Data_Sheet_1.pdf]

Supplementary Material

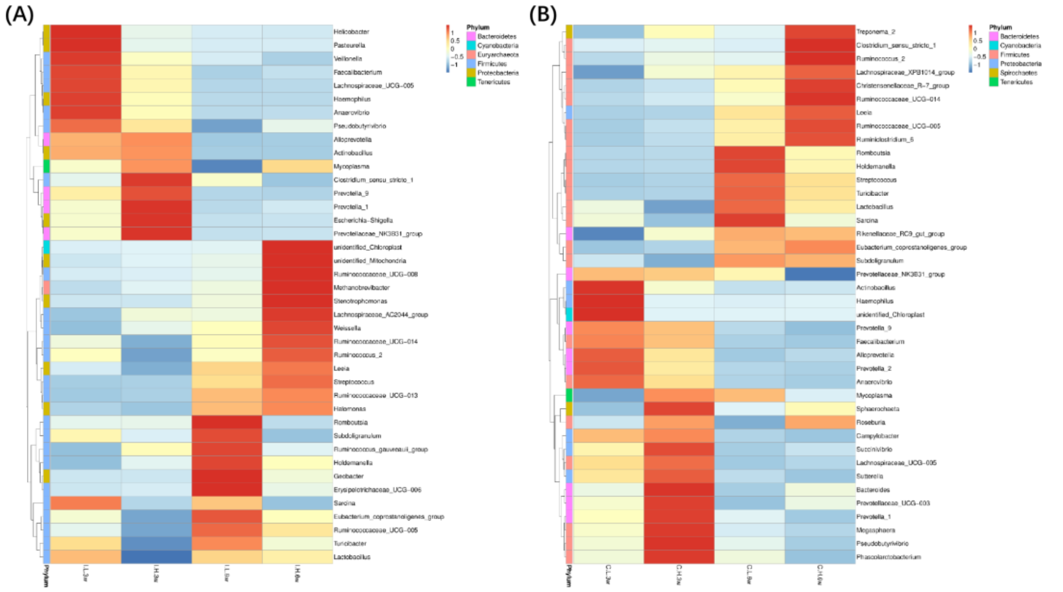

Figure S1 The heat map of top 40 most different genera in ileum and cecum of piglets between control and Cu supplemented groups after 3 weeks and 6 weeks feeding. (A) the heat map of top 40 most different genera in ileum, **(B)** the heat map of top 40 most different genera in cecum. I, ileal microbiota; C, cecal microbiota; L, control group; H, Cu supplemented group; 3w, 3 weeks of feeding after weaning; 6w, 6 weeks of feeding after weaning.

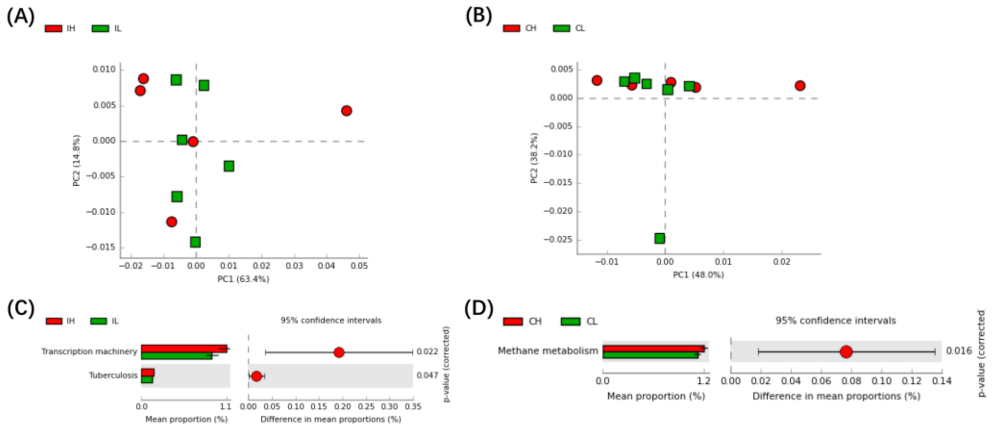

Figure S2 Analysis of functional differences for the ileal and cecal microbiota of piglets between control and Cu supplemented groups after 3 weeks of feeding. (A) PCA analysis for the functions of the ileal microbiota, **(B)** PCA analysis for the functions of the cecal microbiota, **(C)** significantly differential functions of the ileal microbiota, **(D)** significantly differential function of the cecal microbiota. IL, ileal microbiota in control group; IH, ileal microbiota in Cu supplemented group; CL, cecal microbiota in control group; CH, cecal microbiota in Cu supplemented group. Data was analyzed by T-test only due to the minor-changes in this microbiome data.

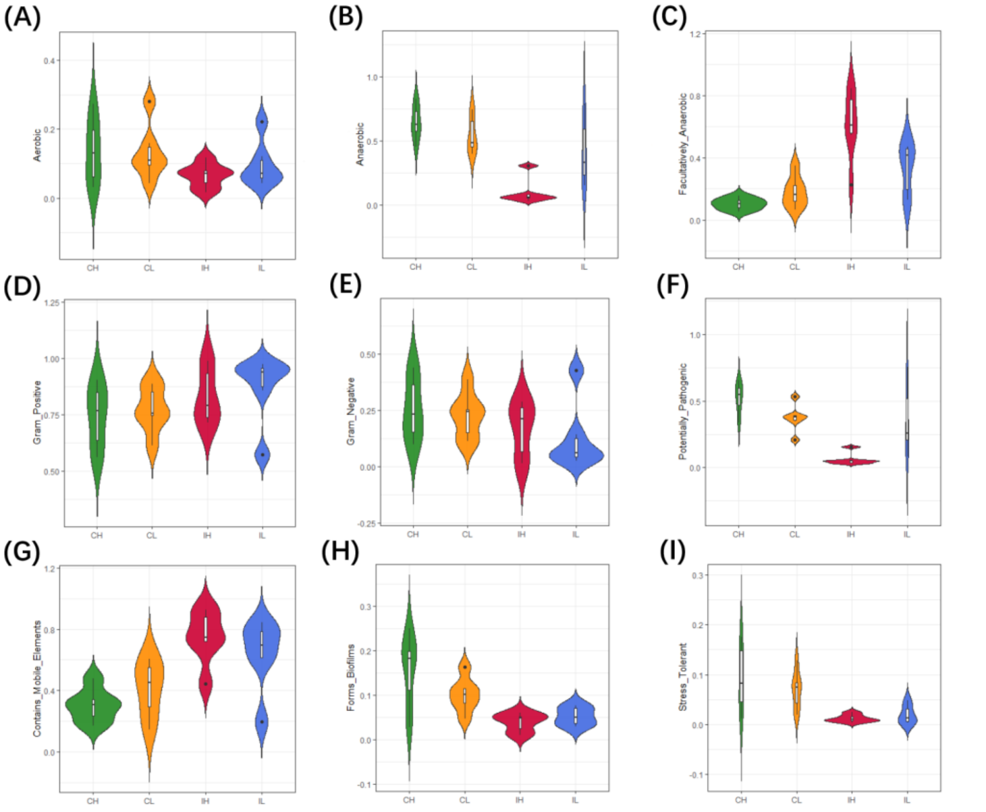

Figure S3 Effects of pharmacological dose of copper on microbial phenotypes in ileum and cecum after 3 and 6 weeks of feeding. (A) aerobic, **(B)** anaerobic, **(C)** facultatively_anaerobic, **(D)** gram_positive, **(E)** gram_negative, **(F)** potentially_pathogenic, **(G)** contains_mobile _elements, **(H)** forms_biofilms, **(I)** stress_tolerant. IL, ileal microbiota in control group; IH, ileal microbiota in Cu supplemented group; CL, cecal microbiota in control group; CH, cecal microbiota in Cu supplemented group.

Table S1. 16S rRNA gene primers used for the detection of *Escherichia coli* isolates

Primer	Sequence (5′→3′)	Length (bases)	Position	Orientation*
16 E1	GGGAGTAAAGTTAATACCTTTGCTC	25	452-476	F
16 E2	TTCCCGAAGGCACATTCT	18	1018-1035	R
16 E3	TTCCCGAAGGCACCAATC	18	1018-1035	R

*F, forward sequence; R, reverse and complementary sequence.

Table S2. Effects of pharmacological dose of copper on α-diversity and abundance of ileal microbiota[1].

Indices	3 weeks			6 weeks		
	Control	200 mg Cu/kg feed	P-value	Control	200 mg Cu/kg feed	*P*-value
Observed_species	370.33	346.00	0.535	450.83	366.50	0.191
Shannon	4.00	3.09	0.149	3.50	3.15	0.473
Simpson	0.84	0.64	0.128	0.73	0.69	0.713
Chao1	466.16	424.78	0.459	494.84	452.20	0.547
ACE	467.35	441.57	0.600	504.91	444.72	0.377
PD_whole_tree	43.70	35.91	0.523	48.16	34.62	0.144

[1]All indices were analyzed by Wilcox test and presented with means. Within a row, values without same small letter superscripts differ significantly (P < 0.05).

Table S3. Effects of pharmacological dose of copper on α-diversity and abundance of cecal microbiota[1].

Indices	3 weeks			6 weeks		
	Control	200 mg Cu/kg feed	P-value	Control	200 mg Cu/kg feed	*P*-value
Observed_species	487.83	473.60	0.714	808.50	957.33	0.266
Shannon	5.75	5.81	0.893	5.93	6.26	0.419
Simpson	0.94	0.93	0.552	0.94	0.96	0.375
Chao1	554.36	549.13	0.907	994.55	1097.23	0.603
ACE	555.11	547.63	0.866	906.74	1098.50	0.235
PD_whole_tree	37.50	37.47	0.994	68.41	88.22	0.176

[1]All indices were analyzed by Wilcox test and presented with means. Within a row, values without same small letter superscripts differ significantly (P < 0.05).

Table S4. Prevalence of antimicrobial multi-resistance phenotypes of *E. coli* in ileum and cecum (%).

Phenotype[a]	Ileum		Cecum	
	Control (n=20)	High Cu (n=30)	Control (n=34)	High Cu (n=35)
None	15.00	13.33	5.88	2.86
SXT		3.33	8.82	11.43
CIP				2.86
C_SXT	35.00	3.33	5.88	11.43
C_AMP	5.00			
AMP_SXT	10.00		2.94	5.71
CN_SXT			2.94	
C_AMP_SXT	15.00	13.33	20.59	17.14
CRO_ATM_SXT		3.33		
C_CN_AMP		3.33		2.86
CN_AMP_SXT			5.88	
AMP_AK_SXT			2.94	
CRO_AMP_SXT			2.94	
CIP_AMP_SXT				2.86
C_CN_AMP_SXT	5.00		5.88	8.57
C_CIP_AMP_SXT		40.00	8.82	8.57
CRO_C_CN_AMP		3.33		
CN_CIP_AMP_SXT			14.71	
CRO_C_AMP_SXT				5.71
C_CN_CIP_AMP_SXT	5.00	3.33	2.94	5.71
CRO_C_ATM_AK_SXT		3.33		
CRO_CN_CIP_AMP_SXT			5.88	
C_AMP_MEM_AK_SXT				2.86
C_CN_AMP_AK_SXT				2.86
CRO_C_CN_CIP_AMP_SXT	10.00			
AMC_CRO_C_CN_AMP_AK_SXT		3.33		
CRO_C_CN_CIP_AMP_ATM_AK_SXT		3.33		2.86
AMC_CRO_C_CN_AMP_ATM_AK_SXT		3.33		2.86
CRO_C_CN_CIP_AT			2.940	

M_MEM_AK_SXT	
AMC_CRO_C_CN_CIP_AMP_ATM_MEM_AK_SXT	2.86

[a] *AMP=ampicillin, AMC=amoxicillin/clavulanic acid, CRO=ceftriaxone, MEM=meropenem, ATM=aztreonam, CN=gentamicin, AK=amikacin, CIP=ciprofloxacin, C=chloramphenicol, SXT=trimethoprim/sulfamethoxazole.*

Table S5. The isolation rates (95% CI) of *Escherichia coli* from anal swabs at d 0, d 21 and d 42 of the feeding period[1].

	Control	20 mg Cu/kg feed	100 mg Cu/kg feed	200 mg Cu/kg feed	*P*-value
0 w	94.44(72.71, 99.86)	83.33(58.58, 96.42)	83.33(58.58, 96.42)	100.00(81.47, 100.00)	0.124
3 w	83.33(58.58, 96.42)	94.44(72.71, 99.86)	83.33(58.58, 96.42)	83.33(58.58, 96.42)	0.651
6 w	91.30(67.64, 97.34)	91.30(67.64, 97.34)	62.50(40.59, 81.20)	95.83(78.88, 99.89)	0.056

[1]*The differences among groups in isolation rates of E. coli were statistically analyzed by Chi square test. The isolation rate of Escherichia coli was defined as ratio of the number of Escherichia coli isolates to the number of all isolates in each group.*
